# Supplementary material for: Spectrum, Time Course, Stages, and a Proposal for the Diagnosis of Histamine Intolerance in General Practice: A Nonrandomized, Quasi-Experimental Study
Source: J Clin Med. 2025 Jan 7;14(2):311. doi: 10.3390/jcm14020311 (PMC11765637; doi:10.3390/jcm14020311)
Supplement: Supplementary file 1 [file jcm-14-00311-s001.zip › Supplementary material 2. Educational material and proposed questionnaire.docx]

**Suppelemntary material 2.**

**Proposed questionnaire for histamine intolerance in primary care**

| **Patient general information** |
| --- |
| Age, Gender, Body height, Body weight, Body mass index |
| Histamine intolerance frequency score (HFS):  1: Symptoms less than 1 day/month  2: 1-6 days/month (1 day/week)  3: 7-14 days/month (2-4 days/week)  4: 15-21 days/month (4-5 days/week)  5: Symptoms occurring mostly every day |
| How severe are the symptoms on a scale of 1-10 (10 is the worst) |
| Leading symptom according to patient  1. Headache  2. Gastrointestinal symptoms  3. Other |
| **Gastrointestinal complaints** |
| Diarrhea |
| Loose stools |
| Bloating |
| Nausea/feeling sick |
| Vomiting |
| Stomach ache |
| Early satiety/dyscomfort |
| Abdominal cramping |
| Dyspepsia/reflux/heartburn |
| Were you prone to recurrent abdominal pain as a child? |
| Have you ever taken medication for reflux/heartburn? |
| **Neurological complaints** |
| Headache |
| Migraine/migraine-like (pulsating, throbbing, half-sided) headache |
| Dull headache |
| Postprandial somnolence |
| Dizziness |
| Benign paroxysmal positional vertigo |
| Commanding or sudden drowsiness (hard to keep eyes open)/postprandial fatigue/postprandial dysphoria |
| **Respiratory complaints** |
| Sore throat |
| A runny nose |
| Coughing |
| Sneezing |
| Has asthma bronchial asthma ever occurred (childhood, adulthood) or asthmatic symptoms (wheezing, dyspnea, dry cough, etc.) where asthma could not be verified |
| Nasal congestion |
| Dyspnea |
| Postnasal drip, throat clearing |
| Any known allergies |
| Recurring upper respiratory symptoms |
| **Skin complaints** |
| Non-extensive hives/urticaria |
| Atopic dermatitis |
| Flush |
| Oedema/swelling |
| Itching |
| Other skin complaints |
| Acne |
| Strong reaction upon insect sting |
| **Cardiovascular complaints** |
| Palpitations |
| Blood pressure drops |
| Near-collapse |
| **Psychiatric complaints** |
| Depression/depressive mood |
| Lack of motivation |
| Brain fog/feeling deconcentrated |
| Mood disturbances |
| Nervousness |
| Anxiety |
| Experiencing a decrease in cognitive processing speed |
| Complaints connected to sleeping |
| **Miscellaneous complaints** |
| Muscle twitching (eyelid…) |
| Weakness/weariness |
| Often recurring herpes labialis/mouth aphthae |
| Acetylcysteine adverse reaction |
| Amoxycillin and clavulanic acid sensitivity |
| Fatigue |
| Experiencing symptoms according to Stage 1 of Figure 2  Experiencing symptoms according to Stage 2 of Figure 2  Experiencing symptoms according to Stage 3A of Figure 2  Experiencing symptoms according to Stage 3B or 4 of Figure 2 |
| Experiencing symptoms according to Timeline 1 of Figure 4  Experiencing symptoms according to Timeline 2 of Figure 4  Experiencing symptoms according to Timeline 3 of Figure 4  Experiencing symptoms according to Timeline 4 of Figure 4 |
| **Anamnestic data** |
| How old were you when the symptoms begun? |
| How many years since symptom onset |
| Are the symptoms intermittent? |
| Onset of symptoms usually in the afternoon or evening |
| Onset of symptoms was as a teenager/young adult (ages 10-35) |
| Headaches or chronic diarrhea in the family |
| Histamine intolerance in the family |
| Someone in the family who took/takes medication because of reflux symptoms |
| I sometime have simultaneous headache with a family member when we are together (common meals) |
| Do your children have any similar symptoms and if yes how old are they |
| I feel more energetic on an empty stomach |
| No symptoms during pregnancy (especially 2nd, 3rd trimester) |
| The onset of symptoms can be attributed to meals |
| **Checklist of the most characteristic symptoms**  **If histamine intolerance (HIT) is strongly suspected, these symptoms should be reassessed** |
| Reflux symptoms (e.g., heartburn) |
| Diarrhea/loose stools |
| Bloating |
| Headache |
| Postprandial drowsiness |
| Fatigue/weakness |
| Symptoms of at least two separate organ systems |
| More than 4 regular, primary symptoms |
| Suspected mast cell activation |
| **Food intolerances** |
| Ketchup, concentrated tomato concentrates, tomato soup; Wine; Champagne; Beer; Sauerkraut/pickles; Coffee (causing e.g., headaches, coughing etc.); Chocolate, Hard cheese (ripened cheese); Tuna; Hungarian sausages/bratwurst/salami/nürnberger/frankfurter; Seasoned potato chips (likely due to seasoning); Citrus fruits; Peanuts; Nuts; Seafood; Legumes (soy, peas, lentils, beans); Strong spices; Milk/milk products (e.g., yoghurt); Scones in oil (Hungarian fast-food); Onions/garlic; Breakfast sausage; Vinegar; Soy sauce; Bakery wares/fast bakery; Eggs; Yeast/yeast products; Other food intolerance |

**Educational material handed out to patients in the study**

| Keep a food-symptom diary! When headaches/diarrhea begin, record the time and the previous 1(-2) meals (and types of fluids)! If the symptoms start in the morning upon waking or during the night, then record the dinner instead! |
| --- |
|  |
| Does my headache usually happen between 0 and 4 hours after meals, except for those that occur immediately upon waking up in the morning? |
| In cases of existing headaches, is it observed that the headache becomes stronger within 0-4 hours after eating? |
| In addition to my headache, do I simultaneously experience diarrhea/loose stools and/or dyspepsia? |
| As headache and dyspepsia may persist for several days, do these symptoms deteriorate upon eating? |
| Do I encounter congestion, runny nose, sneezing, or dry cough during or shortly after eating without any known allergic trigger? |
| Do I find it necessary to clear my throat after having a meal? |
| Do I notice postnasal dripping, irritation in the mouth or throat, muscle twitching (especially in the upper eyelid), or mouth aphthae following certain meals? |
| Can commanding drowsiness, unexplained lethargy, fatigue, nausea, dizziness, palpitation, bloating, early satiety or brain fog be linked to eating on occasion? |
| Do I develop headache, diarrhea or bloating when deliberately consuming a diet that contains histamine-rich food in regular quantities, such as sausages, salami, hard cheese, alcoholic beverages, chocolate, ketchup, food with tomato paste, spicy-food, pickles, citrus fruits, organ meats, certain dairy products, soy sauce, etc.? |
| In the case of a very severe, persistent headache lasting for days, does a diet consisting of water, salt, potatoes, apples, and fresh chicken breast help alleviate the symptoms? |
|  |
| Examples of prolonged histamine-related symptoms that do not appear immediately after eating and thus might cause confusion: acid-related, skin, upper respiratory, psychological, and asthmatic complaints, and dizziness. Diarrhea may be delayed until the next day, and stomach acid complaints may occur at night. |

| **Food-Symptom Diary** | | | | | | | | | |
| --- | --- | --- | --- | --- | --- | --- | --- | --- | --- |
| This table is used to record the foods consumed in the previous 4 hours. Mark where necessary! Headache or acid reflux symptoms can persist for several days; in such cases, observe whether eating WORSENS the symptoms. | | | | | | | | | |
| Onset after eating  🡪 | | 0-30 minutes | 0-2 hours | 0,5-4 hours | 0,5-4 hours | 0-4 hours, sometimes up to 8 hours | 0,5-4 hours, sometimes until the next morning | 0-4 hours |  |
| **Date, time** | **Food consumed** | **Runny nose, nasal congestion, sneezing, post-nasal drip, throat irritation, mouth irritation, eyelid twitching, muscle twitching, early/uncomfortable feeling of fullness** | **Bloating, early satiety, abdominal discomfort, cramps, fullness, abdominal pain** | **Headache migraine** | **Drowsiness after eating, concentration difficulties, tiredness, fatigue, lethargy, brain fog** | **Loose stool, diarrhea** | **Acid symptoms, heartburn** | **Other (e.g. nausea, dizziness, tiredness, skin symptoms, cough, worsening of asthma symptoms, aphta, palpitations, drop in blood pressure, near-collapse, itching)** |  |
|  |  |  |  |  |  |  |  |  |  |
|  |  |  |  |  |  |  |  |  |  |
|  |  |  |  |  |  |  |  |  |  |
|  |  |  |  |  |  |  |  |  |  |
